# Experience with an online positive psychology intervention for caregivers of people with Alzheimer’s disease: an interpretative phenomenological analysis

**DOI:** 10.1080/17482631.2025.2494349

**Published:** 2025-04-28

**Authors:** Marie Dobignies, Clotilde Larochette, Eva Andreotti, Faouzia Millequant-Gourari, Johanna Gonzalez de Linares, Louise Lefebvre, Pascaline Cassagnaud, Florence Pasquier, Pascal Antoine

**Affiliations:** a CNRS, UMR 9193 – SCALab – Sciences Cognitives et Sciences Affectives, University Lille, Lille, France; b CHU Lille, Department of Neurology, Memory Research and Resources Clinic, Lille, France

**Keywords:** Informal caregivers, Alzheimer’s disease, interpretative phenomenological analysis, online support, positive psychology

## Abstract

**Purpose:**

Considering the distress experienced by caregivers, numerous support systems have been devised. Recently, interventions focused on positive psychology have resulted in beneficial effects for caregivers. This study aimed to investigate the experience of caregivers of people with Alzheimer’s disease with an online positive psychology intervention.

**Methods:**

To understand caregivers’ experiences of an 8-week positive psychology intervention, 10 participants participated in a semistructured individual interview. Interpretative phenomenological analysis was carried out on the interviews.

**Results:**

Three themes were identified. [1] Engaging half-heartedly: Positive psychology was initially perceived as an unknown and caregivers expressed their scepticism before gradually integrating the intervention into their daily routine. [2] Letting yourself be destabilized: Caregivers went through an uncomfortable phase, becoming aware of their overprotective or controlling behaviours and the consequences of their psychological distress. [3] A springboard to change: Several realizations resulted in changes, such as reactivating internal resources and cultivating a more balanced outlook with less focus on the illness of the care receiver and the caregiving situation.

**Conclusions:**

The results show that caregivers were completely autonomous during the online intervention and engaged in the processes promoted by positive psychology, which supports further development of online resources for caregivers.

## Introduction

In Alzheimer’s disease (AD), caregivers play a major role in allowing their loved ones to remain in their own homes and to preserve their quality of life (Etters et al., 2008). Therefore, understanding caregivers’ experiences is essential (Galvin et al., 2005). The literature highlights the difficulties faced by caregivers (Bertrand et al., 2006; Ory et al., 1999; G. Smith & Rodham, 2022), including issues relating to their physical health (Capistrant et al., 2012; Von Känel et al., 2008) and mental health (Chiao et al., 2015; Ferrara et al., 2008; Frias et al., 2020; O’Dwyer et al., 2016). These difficulties are influenced by numerous factors, such as caregivers’ personal experiences (Ruyant Belabbas et al., 2024), support resources available (Gilhooly et al., 2016), estimation of cognitive and functional abilities (Nichols et al., 2024) or gender biases involving estimates of the financial capacity of the care receiver (Giannouli & Tsolaki, 2022). From a sociocultural perspective, caregiving is still predominantly undertaken by women. Taking on more tasks over a longer period places them at greater risk of experiencing emotional burden (Gaugler et al., 2005).

To support caregivers, psycho-socioeducational interventions, counselling, and combined approaches have mainly been used; such approaches result in genuine and moderate levels of effectiveness (Cheng & Zhang, 2020; Dickinson et al., 2017; Elvish et al., 2013; Giannouli, 2017; Walter et al., 2020). However, face-to-face interventions are subject to two limitations. On the one hand, they are not always suitable for the issues that caregivers face; exhaustion, lack of availability beyond the caregiving itself, and the need to travel to places of care can be obstacles to engaging in a psychological support approach (Brodaty & Donkin, 2009). More flexible and less restrictive solutions, especially those that are online, are warranted to complement existing solutions. On the other hand, while providing assistance is a source of positive experiences (Lloyd et al., 2016; Netto et al., 2009; Peacock et al., 2010; Sanders, 2005; Yu et al., 2018), positive experiences are often overlooked relative to interventions that address stress regulation and burden alleviation. Developing programmes that support the positive dimensions of caregiving could provide caregivers with a sense of self-efficacy and personal growth, a better quality of life, and an improved relationship with the care receiver (Carbonneau et al., 2010; Netto et al., 2009; Quinn et al., 2019).

Thus, this study was part of a process to build a new support system that is complementary to existing interventions and that focuses on two characteristics: supporting the positive dimensions of the caregiving relationship and being available online.

Regarding the integration of the positive dimensions of the caregiving relationship, the intervention consists of an 8-week positive psychology programme. The principles of positive psychology were described by Seligman and Csikszentmihalyi (2000) based on their reflections on the value of cultivating personal strengths and resources. Positive psychology was further defined as “the study of conditions and processes that contribute to the development or optimal functioning of individuals, groups, and institutions” (Gable & Haidt, 2005). Closely linked to the concepts of well-being and autonomy, positive psychology interventions (PPIs) integrate cognitive and behavioural exercises regarding the themes of kindness and gratitude, as well as creativity. In work conducted in general or clinical populations, the main results have highlighted the benefits of PPIs for well-being (Bolier et al., 2013; Hendriks et al., 2020; Hone et al., 2015; Page & Vella-Brodrick, 2013; Sin & Lyubomirsky, 2009) and the development of positive feelings, behaviours, and emotions (Cohn & Fredrickson, 2010; Lambert et al., 2019; Lyubomirsky et al., 2005; Sheldon & Lyubomirsky, 2006). More recently, research has highlighted the effectiveness of PPIs with diverse caregiver populations (Chen et al., 2020; da Silva & Giacomoni, 2020; Nevin et al., 2022), but few studies to date have explored the value of PPIs with AD caregivers. Interventions specifically aimed at increasing positive emotions can be expected to have beneficial effects for psychological distress, perceived stress, and depressive symptoms for caregivers of people with frontotemporal lobar degeneration (Dowling et al., 2014; Verstaen et al., 2018). A study of caregivers assisting family members with neurodegenerative disease highlighted increased positive emotions, improved perception of the positive aspects of caregiving, and improved mental and physical health (Moskowitz et al., 2019). In addition, the increase in positive emotions appears to mediate the effect of the intervention on depressive symptoms. This promising work on positive emotions encourages broader development of PPIs that account for each of the five dimensions of the PERMA model (Seligman, 2011): positive emotion as well as engagement, relationships, meaning, and accomplishment (Antoine, 2022). Thus, research is needed, on the one hand, to develop PPIs specifically tailored to caregivers of people with AD and, on the other hand, to better understand the experience of caregivers and identify the processes of change during a PPI.

Regarding the use of an online format, this type of intervention is intended to be readily deployed over a large geographical area and thus reach many caregivers. Thus, an online device is also expected to be suitable in a variety of personal and economic circumstances. The Internet provides a substantial cost‒benefit ratio: it allows better accessibility of psychological interventions (Hollis et al., 2015; Liu et al., 2022), sustainability through the use of an online site, the use of a variety of digital media and the convenience of being at home (Mitchell et al., 2010), user empowerment, and an active role in behaviour change processes (Walsh et al., 2018). In the past decade, in general and clinical populations, the increasing proportion of people likely to use the Internet has led to the development of online PPIs (Sitbon et al., 2019). In the clinical population, these interventions have been reported to provide the same benefits as those delivered face to face, such as improved well-being (Gander et al., 2013; Mongrain & AnselmoMatthews, 2012) and the reduction of depressive symptoms (Proyer et al., 2014; Schueller & Parks, 2012). However, AD caregivers’ experience of online interventions has yet to be documented.

The objectives of the study stem from two challenges. The main objective was to explore the experiences of a positive psychology programme by caregivers of a person with AD or a related disorder (dementia with Lewy bodies; frontotemporal lobar degeneration; vascular and mixed dementia). More specifically, our study aimed to understand caregivers’ experience of the programme as a whole and the activities offered, as well as the meaning given to their progression during the support programme. The secondary objective concerned more specifically how this type of support was delivered. Our study focused on the caregivers’ experience with remote support based on proposals for activities focused on well-being and the integration of this digital tool into their daily lives. It also addressed issues of the accessibility and acceptability of the proposed support.

To address these two objectives and to access the experiences of caregivers, the meaning they assigned to their experience and the changes they observed, a qualitative method was selected (Dovi et al., 2021; Durepos et al., 2019).

## Method

### Procedure

This study was an ancillary qualitative study of intervention research. Participation in this study was voluntary and required signing a consent form. The participants were assured of the confidentiality of the datA.

A positive psychology programme was integrated into an online site created in 2019 for caregivers of people with AD or a related disease. This website offers four independent programmes, each lasting eight weeks, with one based on mindfulness, one on acceptance and commitment therapy, one on information and one on positive psychology; this study focuses on the last programme. The participants were randomly assigned to a programme. Without explaining the theoretical underpinnings of the proposed approaches, the participants were informed of the programme’s main objectives and themes.

The positive psychology programme consisted of 48 activities (Table I), each of which was based on one of the five dimensions of the Positive Emotion, Engagement, Relationships, Meaning, and Accomplishment (PERMA) model (Seligman, 2011) and was aimed at promoting individual development (Antoine et al., 2020; Congard et al., 2020). A detailed outline of the intervention content is in the Appendix. The activities were divided in equal proportions into three areas: the well-being of the participant themselves, the well-being of the participant in their relationship with the care receiver and acquaintances regardless of the disease, and the well-being of the participant in their relationships of caregiving with the care receiver and acquaintances. The caregiver had access to a new activity every day for eight weeks, including six activities to be carried out per week and a “rest day”. The activities lasted from 5 to 20 minutes. From the moment the participant started the programme, regular contact was made by phone to ensure there were no technical issues. Testimonials inspired by feedback from caregivers during previous psychological support systems were also provided to the participants throughout the programme. At the end of the eight weeks, the programme ended, and the caregiver was contacted again to carry out a semistructured qualitative interview.

**Table I.** Overview of the content of the positive psychology programme.

Dimensions	Name of activities	Objectives
Positive emotions	•Three little things•Savour and capitalize•A touch of gratitude	Activate a memory of positive events related tocaregiving, capitalize on these positive events, and be thankful for the positive things in life
Engagement	•Identifying your strengths•Using your strengths•Building up strengths•Metaphor of the tree of life	Identify personal strengths related to the caring situation that can be used on a daily basis to increase satisfaction and pleasure
Relationships	•Kindness•Positive words for those around you•Letting go of moments of frustration•Forgiveness•Codeveloping and maintaining links Life story: past, present, future	Cultivate the feeling of being connected to others and to the person being helped; note the links with one’s actual experience.
Meaning	•SharingTransmission•Activities that have meaning•Shared activities	Feel connected to one’s life direction and the meaning assigned to the caregiving
Accomplishment	•Self-care activities•Creating a nice moment for yourself•Two aspects of help: giving and receiving•Changing a habit•The Optimistic Self•Giving time to yourself	Develop a sense of satisfaction through activities that are important to you

### Participants

The inclusion criteria were as follows: (i) being a caregiver (spouse, child, sibling, or friend) of a person with AD or a related disorder; (ii) being over 18 years of age; (iii) being physically and cognitively able to communicate in French with a researcher; and (iv) having daily access to the Internet. Between September 2019 and December 2021, 45 caregivers recruited in memory consultation centres in Hauts-de-France started the positive psychology programme.

Twenty-nine of the included caregivers participated in the end-of-programme visit, and 23 volunteered for an interview. The first ten interviews were analysed. Eight of the caregivers were women, and the mean age was 57.80 years (SD = 16.16). The mean time between diagnosis and maintenance was 30 months (SD = 25.35). Half of the caregivers were caring for a parent, and the other half were caring for their spouse. The caregivers carried out 74.9% (SD = 21.1) of the 48 activities (Table II).

**Table II.** Participant information.

Caregiver^1^	Age of the caregiver	Person with the disease	Diagnosis	Disease duration (months)	Lives with the person being helped	Percentage of activities completed
Alice	69	Mother	AD	60	No	98
Béatrice	63	Spouse	DLB	30	Yes	46
Chloé	59	Spouse	FTLD	24	Yes	33
Dorothée	60	Spouse	AD	14	Yes	77
Emilie	25	Father	AD	2	No	94
Fiona	66	Mother	AD	84	No	65
Gabriel	43	Mother	AD	24	No	77
Hélène	43	Mother	AD	2	No	83
Isabelle	72	Spouse	AD	36	Yes	88
Jérémy	78	Spouse	FTLD	24	Yes	88

1. Pseudonyms.

DLB: Dementia with Lewy bodies; FTLD: Frontotemporal lobar degeneration; AD: Alzheimer’s disease.

### Data analysis

The interview and testing process followed the guidelines for interpretative phenomenological analysis (IPA) (Antoine & Smith, 2017; Nizza et al., 2021). IPA was preferred over other qualitative approaches (e.g., grounded theory, thematic analysis) because our objective was largely to understand the experience at the individual case level. We tend to focus on caregivers’ experience of the positive psychology intervention, including their perceptions and views of the particular phenomena that occur (Smith et al., 2022). Previous research has used IPA (Smith & Osborn, 2003) to explore the individual and dyadic experiences of caregivers of people with AD (Clare et al., 2005; Mwendwa et al., 2021; Quinn et al., 2019; Wawrziczny et al., 2016). This approach makes it possible to capture the singular experiences of participants and the meaning they assign to these experiences. Based on a double hermeneutic, it addresses, on the one hand, the participant’s understanding of their own experience and, on the other hand, the researcher’s interpretation of the interviewee’s experience. IPA requires a relatively small and homogenous sample for greater depth of analysis (Smith & Rodham, 2022). Since the aim was to understand participants’ experience with the programme and not the disease, the criterion for homogeneity was to have followed the programme through to the end, even if not all the activities were completed. The interviews are generally conducted face-to-face and individually to promote a reflective approach, but an alternative involving being interviewed by phone is possible and an option depending on the circumstances (Antoine, 2017; Turner et al., 2002).

In this study, the analyses focused on the caregiver’s experience with positive psychology and online access. They were based on what the caregiver revealed regarding their experience of the positive psychology programme in their own words. The interviews took place at the caregiver’s home, except one participant who requested a personal communication The interviews were semistructured and focused on the following topics: the participants’ expectations regarding the programme, any difficulties encountered, the way in which the digital tool had become part of daily life, and changes observed according to individual and relational dimensions of aid. The recorded interviews were fully transcribed.

To enhance the reflexive process of the interpretations, four psychologists trained in IPA or psycho-gerontology took part in the analyses and were supervised by an IPA instructor. Following previous IPA studies with a caregiver population attending a neurodegenerative disease (Constant et al., 2022; Manceau et al., 2023; Ruyant Belabbas et al., 2024; Wawrziczny et al., 2016), the psychologists went through the following steps: the first interview was read several times, who cross-referenced experiences as much as possible. Each psychologist annotated the interview independently of the others to identify the salient elements of the participant’s feedback, contradictions, and the progression of the discourse going back if necessary. At the end of this phase of individual analysis, the psychologists shared their findings, first as an overall perspective and then as a linear review of the entire interview point-by-point to collectively enrich the salient points and interpretative directions. All these elements were then outlined schematically in the form of a mental map synthesizing the collective analysis. This diagram was presented and explained to the supervisor, whose role was to check the consistency of the analyses and question the interpretations regarding aspects of the interview that remained unclear, any discrepancies between the diagram and the explanations, and—if needed—ask for a new stage of analysis and further elaboration of the schematic outline. This process was then repeated for each interview and presented to the supervisor. Once the ten interviews had been analysed, the psychologists met and studied all the diagrams to retain the main themes and identify the common elements, as well as the interpretations concerning only a minority of interviews. Based on the themes thus identified, all the interviews were reviewed to collect extracts that illustrated or qualified these results and provide an overview of their density within the sample.

## Results

The interviews capture how the relationship of caregivers with the positive psychology programme evolved over the eight weeks. While people’s experiences may have been tinged with a degree of reticence in the beginning, the hope aroused by the programme and its integration into daily routine allowed this reluctance to be overcome. Different realizations, sometimes painful, were experienced and were accompanied by changes. Beyond alleviating the feeling of loneliness, some caregivers developed more balanced perspectives of caregiving and interactions with the care receiver, taking the initiative, and reactivating personal resources.

### Engaging half-heartedly

Both curious about a new intervention and perplexed about what positive psychology, which was previously unknown to them, could provide them, several caregivers (Hélène, Jérémy, Dorothée) reported their initial reluctance to engage in such a programme.

**Why get help when so far, so good?** Some doubts were based on having only a moderate need for help: the disease was deemed to be at a mild stage, and the symptoms were not experienced as invasive, so caregivers were able to maintain daily life in a way quite similar to that prior to the diagnosis. Jérémy is reluctant to log in every day for 20 minutes and to incorporate the online system into his daily life.

**Jérémy**: Up until now, I had a certain feeling, but I would have a much more pronounced feeling if my wife’s condition had deteriorated […] we have a more or less normal life […] I even saw the program as a constraint. I thought, “Am I going to find 20 minutes every day to devote to this?”

For some caregivers, the context of the study, which was conducted during the COVID health crisis, reduced or even eliminated physical interactions with the care receiver.

**Hélène**: I’m not at the stage to answer these questions. Because my mom, at the moment she’s in the mild Alzheimer’s stage, so the relationship I have with her right now, because of COVID, is by phone. I rarely see her, and when we do meet up, we do everything to “pause” her illness; we do not talk about the things that are upsetting.

However, this low level of need is partly contradicted in these excerpts, as the caregivers implemented measures to avoid negative interactions or already had difficulty finding a few free minutes daily at the stage of mild illness. Moreover, these statements are paradoxical because while worsening of the disease was anticipated, these caregivers maintained the status quo and did not talk about steps to prepare for the future.

**I will try it anyway**: Another point of resistance concerned the gap between the proposed online activities and the caregiving situation. At the beginning of the programme, Gabriel mentioned questioning the potential benefits he could derive from it or the very possibility of integrating positive psychology into his daily life.

**Gabriel**: I had a little trouble sometimes, depending on the exercises. I didn’t think they were…. I had trouble making the link with the support of a loved one […] and then by encountering situations, “Oh well, actually”, I was able to make the link with what we had said before, what had been done in activities. That’s it, the support, the links are made a posteriori.

The adoption of the programme was, therefore, gradual for several participants. While Gabriel doubted the very nature of the activities and then came to realize their relevance a posteriori, others, such as Dorothée, understood the way the programme worked as a structuring factor, a form of personal discipline. Compliance with the proposed framework was sufficient initially to provide a degree of accomplishment.

**Dorothée**: I said to myself “I have to do it, I have to do it in the morning”, and that was it; we feel happy to have done it […] I like it. I’m thorough, so you have to be strict about it. For example, doing it at eleven o’clock in the evening and doing it the next day at nine o’clock in the morning, it makes no sense.

Still others chose to trust and were actively engaged, driven by their open attitudes, as well as by the hope of well-being and of being able to rely on any new resource in light of the difficulties that arise.

**Béatrice**: Compared to everything I’m currently experiencing, everything is a consideration, everything should be tried, in an effort to be better. I was completely up for it. Plus I like new experiences; I like it. I’m quite open to all that, so it was really a plus.

Engagement in the programme did not appear to be based on specific expectations related to positive psychology or the theme of well-being. Indeed, the caregivers discovered this type of content and initially invested themselves in reflecting on their personality, the difficulties experienced, or their life paths, such as Fiona, whose artistic talents fit well with certain activities.

**Fiona**: There is an activity where I had gone off into my artistic field, I had let go a little. All at once. I thought, “Oh no, no, I still have to do something serious” (laughs) when I had to make the tree with the leaves. I had gone into something, I was making a painting, I was gone… with my colored pencils.

### Letting yourself be destabilized

While the initial goal of positive psychology is to promote well-being, the caregivers initially went through a phase of destabilization. This phase can be described to different degrees in relation to the personal itineraries of the caregivers.

**Facing your own barriers**: Jérémy’s words shared after the first few days of the programme describe the general experience of self-reflection and questioning favoured by the programme quite well.

**Jérémy**: There are certain questions that force you to go… deep enough in yourself if you want to be honest with your answers […] it still forced me to question myself, consciously or unconsciously.

Through concrete and brief activities, the majority of the caregivers became aware of attitudes or behaviours that came about gradually. As the disease progressed, some caregivers engaged in overprotective or controlling behaviours or tried to take on increasing responsibilities related to the disease on their own. Isabelle, a caregiver, explained that she was regularly corrected by her daughters—“that is not how you are supposed to handle dad” – and that the programme helped her better assess her actions and behaviour. Identifying these behaviours and their consequences for oneself in terms of exhaustion, isolation, or feelings of helplessness then allows one to modify them, as Gabriel explained.

**Gabriel**: We set up home help. It was also done in parallel, and personally, I am, I get annoyed less quickly. I manage to take a step back […] and then I do not feel like I’m… I admitted that I should not do everything on my own. It was a bit complicated […] and to think about it and extract the positive points, the negative points, and correct, ultimately it is not about correcting, it is to improve, rather, to optimize the support, and to know how to take a step back.

For some like Chloé, the programme was experienced as a learning process, an important and overall assessment of perspectives of oneself, of the relationship, and of the loved one themselves.

**Chloé**: Things to learn, things to understand about yourself, about sharing, about the things you do with the care receiver… In addition, become self-aware… Self-awareness because it is truly about being… regaining self-awareness, that we forget ourselves anyway.

**This showed me my shortcomings**: While positive psychology activities can promote changes in the long term, the immediate experience can be painful for some participants, especially when the programme is interpreted as highlighting a disability of the caregiver or when the questioning induced is intense and confronting, even possibly guilt-inducing.

**Béatrice**: I couldn’t find the three things [editor’s note—the three good things activity] … And I think that’s a little bit my problem. I can’t tell myself that life is beautiful… I couldn’t. At first it worried me; it made me uncomfortable. I take care of him body and soul… But I can’t say I’m having a blast. Love… Love is still there, but it’s not the person I knew, married, lived with for 30 years… The new person who is currently there… it’s more of a burden.

Béatrice pointed out several phenomena, including an awareness of not being able to identify positive aspects in everyday life or to rely on positive aspects of the relationship with her afflicted spouse, whom she no longer recognized as her husband. Activities that invite the caregiver to find the positive in daily life, even if fleeting, or to maintain their original link to the person being helped can be offset by an incompatible experience. Some caregivers give up activities (Béatrice, Chloé) when they do not correspond to their functioning or when the issues are too current and intense.

**Emilie**: There is a passage in the program, a story of forgiveness… I didn’t really explain why I was angry and how I felt, I just marked [editor’s note—daily feedback on the platform] that it was in relation to my sister. But… Forgiveness is a little more complicated […] I am disappointed, I have two sisters, and they did not engage in the program. They still have their lives outside of this, as I am told so well. But they could have taken time to do it, and I think that would have been important.

In Emilie’s case, the conflict with her sisters, with whom she shares the help, was too intense for her to question it or to reconsider her attitude. While talking about her preference for a mindfulness group and her difficulties in adopting the positive psychology programme, she wanted her sisters to do it, as she believed that certain activities would be relevant to them.

### A springboard to change

These realizations were associated with changes in perspectives about oneself, including about one’s own loneliness and suffering but also about the care receivers and interactions. The participants took the initiative, and behavioural changes became possible.

**Overcoming the loneliness**: Several phenomena contributed to a decrease in the caregivers’ feelings of loneliness (Jérémy, Dorothée, and Béatrice). For Jérémy, being offered the programme meant that professionals were taking his situation into consideration and supporting him. For Dorothée, the breakdown of the feeling of loneliness was even stronger. She felt driven by the realization that she was not the only one in this situation of providing care. Inspired by the examples given by the avatars of fictional caregivers, she felt encouraged to take the initiative and to solicit her acquaintances and her neighbours to receive support. Finally, she appropriated the platform in a novel way in moments of isolation at home to express herself as in a logbook or a personal diary.

**Dorothée**: Feeling less alone, in relation to my husband, in relation to the disease… It’s not just me, everyone has to help their loved one […] Being helped morally, being more positive, because the little characters [editor’s note—avatars of fictional caregivers] often talked about it; they said “oh yes, my neighbor helped me”…, going to seek comfort sometimes from others. And it feels good to talk about it; since I can’t always talk about it to everyone, I was talking about it on the computer.

**Knowing how to recognize the positive**: After becoming aware of the situation experienced, some positive psychology activities allowed for a change of perspective with an impact on everyday life and the relationship. Unlike Béatrice, who was challenged by the three good things exercise, Fiona reported that this activity allowed her to recognize her suffering and her essentially negative view of everyday life. She did not feel compelled to see the situation positively but rather felt empowered to take a more complex look at situations. She explained that she had developed a fairly effective skill of gaining a new perspective. This required a voluntary effort and necessitated a slight delay compared to the situations encountered.

**Fiona**: I was very down. I was not well, and it made me realize that even in bad times, there could be good things anyway. It sometimes allows me to take a step back, when I feel that things are not going well or that I resent them a little for certain things. Maybe not immediately, but with, a few minutes or, not even a few hours, one hour maximum. Where I say to myself, “No. I have to think about something else. Isn’t there something that is better and see what can be good in all this?” I try; it’s not always easy, but I find that it works quite well anyway. For me, it was really important, because it’s true that I couldn’t see what was good.

**Perceiving my loved one positively again**: This skill marked a break from before the programme. For Hélène, in addition to the ability to take a step back, it was the perception of the care receiver that was altered by the activities and that reduced the suffering she felt, at least temporarily.

**Hélène**: We ask ourselves, it’s well done, we manage to see the person and not the disease, to see what it has brought us … And it can really help us when we are a little bit in mental distress. It feels good; it’s a breath of fresh air.

Thus, the exercises allowed the caregivers to reconnect with the care receiver and to distance the focus on the disease. This change in attitude was accompanied by behavioural change and led to less stressful interactions.

**Bolstering resources**: The positive psychology programme gradually became a springboard to action. Some caregivers felt liberated and more confident, as Alice said. They used the programme to mobilize internal resources and improve their skills related to caregiving. Positive psychology became a reinforcer, reassuring caregivers regarding what they wanted without daring to implement it.

**Alice**: Being aware that I was limiting myself, and in my own head: “I would like to do this project”; before it was, “Oh no, I will not succeed, there are limitations, it is not possible”. There, it’s like an awakening inside me, and then it’s possible! […] As I dared to do things, to approach people, it put a little bit of euphoria in my head: “Hey what are you doing here? What’s going on?” I felt changed […] I was strengthened when I performed a specific action, “it’s okay, it works!”

## Discussion

This study examined the experiences of caregivers of people with AD or related diseases in an online positive psychology programme. The main objective was to explore the experience of caregivers and the meaning given to this experience and to understand the processes activated by positive psychology activities. The secondary objective was to explore caregivers’ experience with support in a digital format. Three major themes were identified.

The theme “engaging half-heartedly” suggests a certain perplexity of the caregivers regarding the proposed online intervention, despite a degree of interest in and curiosity about the programme. The results highlight two types of reluctance, one related to the caregivers’ difficulties in using support services and the other related to the value of integrating positive psychology into their daily lives.

First, the caregivers exhibited a degree of hesitation to accept and engage in the process. These results are in line with the literature, which shows that, irrespective of the stage of the disease, caregivers have difficulty appropriating the proposed aids (Leocadie et al., 2018; Neville et al., 2015). They adopt avoidance strategies, minimize symptoms, prefer not to talk about the disease, or talk about the future. In the early stages of the disease, many spouses or children do not see themselves as caregivers, which can lead to difficulties in identifying a need for support (Wawrziczny et al., 2016). At a more advanced stage, the constraints related to the exacerbation of the disease lead caregivers to no longer allow themselves moments of respite (Quinn et al., 2008).

A second reluctance concerns positive psychology. Indeed, the caregivers were not aware of the concepts of positive psychology and were, therefore, quite sceptical about the relevance of integrating this approach into their daily lives. Nevertheless, the framework inherent in the programme proved to be reassuring to the caregivers. Aside from positive psychology activities, the caregivers responded initially to a need to feel accompanied, supported, and thus overcome the loneliness associated with providing assistance (Ducharme et al., 2009; Wawrziczny et al., 2018). Following the second objective, the online format was also generally accepted: for some caregivers, it was a new experience that differed from more traditional supports, while other caregivers appreciated the ease of use of the site and the option to carry out the activities without time or travel constraints. The literature reports similar results regarding the accessibility and usability of online approaches (Dowling et al., 2014; Hollis et al., 2015).

Following this commitment to the programme, the second theme, “letting yourself be destabilized”, highlights a difference between the suggested positive psychology activities and the actual behaviours of the participants. Indeed, while the programme promotes activities focused on behavioural activation and well-being, caregivers first go through a stage of questioning. Realizing that they had not previously behaved in a manner that the programme suggests that they try, the caregivers felt that they had missed out on certain experiences or ways of approaching situations. This awareness could explain the gradual realization of the benefits related to positive psychology, described in the third theme. This result may also reflect the seminal study of Seligman et al. (2005), which showed that some positive psychology exercises that promote action take several weeks to become effective.

For the majority of the caregivers, the questioning phase resulted in a feeling of astonishment and destabilization. The participants were able to become aware of certain attitudes that had exacerbated their exhaustion and feelings of loneliness. By experimenting with new behaviours or new ways of seeing their experiences as caregivers, the participants were able to overcome certain barriers and set the stage for change.

However, for two caregivers, the feelings related to this phase of introspection were more intense, associated with a feeling of guilt or anxiety related to the fear of failure of the proposed exercises. Two activities in particular reflected current tensions between the participants and their family or professional acquaintances. These results highlight the need to adjust the activities that are proposed when devising a standardized programme, in particular through the creation of modules of activities involving progressivity. One consequent recommendation could be the development of activities that initially promote positive emotions and then, in a second stage, activities that focus on more complex emotions involving those around them and potentially tense relationships.

Finally, the third theme, “a springboard to change”, describes how, over the course of the programme, the caregivers relied on an awareness of their difficulties in engaging in a process of change.

A surprising result, which does not appear to be specific to positive psychology but touches on the second objective of the study, was to overcome loneliness. Indeed, the online format was not primarily intended to reduce feelings of isolation. However, some caregivers considered the online tool a device that accounts for caregivers, their needs, and their constraints. The study on the online resource developed by Dowling et al. (2014) with caregivers of people with frontotemporal lobar degeneration led to similar conclusions. In addition, most caregivers in our study appropriated the tool to virtually share their feelings and emotions during moments of solitude. The invitation to log in daily and record a feeling about the activity often was reinterpreted as an opportunity to use the online site as a logbook. Testimonials to illustrate the exercises were also a source of support and normalization of difficult feelings.

Our study also highlights the progression of caregivers regarding their ability to perceive positive aspects of caregiving situations. This result is in line with previous research, notably that by Moskowitz et al. (2019) of an intervention specifically focused on positive emotions with caregivers of people with AD. More specifically, their work highlights a significant increase in positive emotions, a decrease in depressive and anxiety symptoms, and an improvement in the ability to perceive the positive aspects of caregiving.

The use of positive reinterpretation skills is also a result consistent with previous studies (Antoine et al., 2018). It brings to mind the *broaden-and-build theory* developed by Fredrickson et al. (2004), which suggests that positive emotions encourage the individual to explore new strategies for changing perspectives. Thus, in our study, better abilities to reassess the events experienced allowed the caregivers to, on the one hand, bring about a change in perspective and restore their identity and, on the other hand, to promote a reconnection between themselves and the care receiver.

Finally, the programme invites participants to identify and mobilize certain personal strengths in concrete actions that promote the development of positive emotions. As a result, these behaviours reinforce the caregivers’ sense of confidence in their abilities to develop caregiving-related skills (Fredrickson et al., 2004; Wawrziczny et al., 2021). and a feeling of being supported in their role as caregivers (Peacock et al., 2010).

### Strengths and limitations of the research

Our study provides a novel contribution, as research conducted regarding online PPIs tailored to the caregiver population is still poorly developed. Our study is also the first to explore caregivers’ experiences with PPIs and the meaning given to this experience. Based first on the participants’ statements, the qualitative approach taken through IPA revealed the mechanisms throughout the programme and the progression of the caregivers in their journey. It was thus possible to more precisely explore the processes found in other forms of psychological support, in particular the reluctance of caregivers regarding assistance in general, and the processes more specific to positive psychology, in particular the reassessment of situations of caregiving and the identification of resources.

However, several limitations can be highlighted. First, the sample consisted mainly of caregivers who were recruited from a centre with expertise in neurodegenerative diseases and who were providing care for a person with advanced AD. The caregiving spouses in our sample were relatively young (60—78 years of age) and not representative of the caregiver population overall. It would, therefore, be interesting to offer this programme to older caregivers, who tend to be more isolated or who are not in a care and support network. Use of the intervention as a prevention tool could be evaluated, particularly for caregivers caring for a person at an early stage of the disease. Moreover, although Alzheimer’s disease affects more women than men, half of the care receivers in our sample were men. Therefore, it would be relevant to include a more balanced sample, allowing us to determine whether the experience of caregivers significantly differs based on the gender of the person cared for.

Additionally, we have created a homogeneous sample in terms of programme completeness. However, the homogeneity can be discussed, since two participants completed less than 50% of the activities and the disease duration varied. Although the analysts did not perceive this diversity of the sample with a significant impact on the results, given that we explore the experience of an intervention for caregivers in their diversity, homogeneity cannot be totally guaranteed.

Collecting feedback only from caregivers who volunteered to carry out the post-test is another limitation of our research. Indeed, we did not conduct qualitative interviews with caregivers who left the study early. However, it would have been relevant to understand the issues that led them to leave the programme as well as to collect their input on the nature of the proposed activities and access to the tool in its digital form.

Finally, the project spanned several years, including two years during the height of the COVID-19 pandemic, with the first caregivers participating in the programme at the peak of the health crisis. This context may have impacted our results concerning the decrease in the feeling of loneliness, especially in a period that limited caregivers’ social interactions and accentuated their isolation (Y. S. Chen et al., 2022).

### Clinical and research implications

To extend this research, further work is needed on caregivers’ experiences of supportive interventions. Indeed, this PPI was tested in an experimental setting with a small number of participants. In terms of accessibility, the caregivers were able to readily use the digital format. These results encourage the proposal of an implementation study that integrates health professionals so that they can readily make use of the proposed tool and act as conduits for caregivers.

The results highlight the potential of the programme, and consideration should be given to its clinical relevance according to the stages of AD. More specifically, the programme appears to be rather suitable for caregivers of a person at an early stage of the disease, when exhaustion is already likely and when a degree of adaptation of behaviours would be useful. On the other hand, deployment of the programme at a time of significant exhaustion may be too late and inappropriate if the caregiver no longer has the mental capacity to benefit from it. More detailed indications, supported by clinical experiments, could guide professionals and promote the most appropriate support for caregivers. Further development of this type of intervention is also promising, in particular by offering more progressive and modular activities and by allowing caregivers to choose activity programmes according to their desire or their personal digital equipment.

Finally, further analyses in terms of effectiveness are needed. Indeed, it also seems relevant to conduct quantitative analyses to observe the potential effects of the PPI on caregivers of people with AD or a related disease.

## Conclusion

The results of this study support caregivers’ positive reception of the programme and identify novel elements regarding the experience of a PPI delivered online in this context. The first theme highlights the necessity of developing psychoeducation to introduce PPI and its various concepts. The second theme emphasizes the need to support participants as they undertake a programme that may be destabilizing. The third theme reveals caregivers’ ability to engage with an online PPI. These themes encourage further development of digital tools complementary to face-to-face support for people caring for individuals with neurodegenerative diseases.

## Data Availability

The data that support the findings of this study are available from the corresponding author upon reasonable request.

## Appendix. Detailed outline of the positive psychology intervention content

**Table:**

Week	Name of the activity	Focus	Description of the activity
Week 1	Day 1. Three little things	The participant	Notice and describe three aspects of your daily life that bring positive emotions and contribute to making your day more enjoyable
	Day 2. Three little things	The participant in their relationship with the care receiver or acquaintances, regardless of the disease	Notice and describe three aspects of your daily life that bring positive emotions towards the care receiver.
	Day 3. Three little things	The participant in their relationship of caregiving with the care receiver	Notice and describe three aspects of your daily life that bring positive emotions towards the caregiving situation.
	Day 4. Self-care activities	The participant	List, plan, and implement simple and enjoyable activities in your day.
	Day 5. Identifying your strengths	The participant	Identify your own skills that can help you feel good, cope with difficult situations, or achieve a goal.
	Day 6. Using your strengths	The participant in their relationship with the care receiver or acquaintances regardless of the disease	Implement new behaviours aligned with your own strengths in your relationship with the care receiver.
Week 2	Day 7. Kindness	The participant	Identify acts of kindness performed during the day.
	Day 8. Kindness	The participant	Plan acts of kindness to carry out during the day.
	Day 9. Kindness	The participant in their relationship with the care receiver or acquaintances regardless of the disease	Identify acts of kindness performed for the care receiver.
	Day 10. Using your strengths	The participant in their relationships of caregiving with the care receiver	Implement new behaviours aligned with your own strengths, in your relationship with the care receiver.
	Day 11. Savor and capitalize	The participant in their relationship with the care receiver or acquaintances regardless of the disease	Expand on a positive event by thinking back on it, reliving it, and continuing to focus on it.
	Day 12. Creating a nice moment for yourself	The participant	Plan a pleasant moment for yourself, ranging from a few minutes to a few hours.
Week 3	Day 13. Metaphor of the tree of life	The participant	Summarize the various concepts discussed (three little things, strengths, self-care activities) through the metaphor of the tree of life.
	Day 14. Using your strengths	The participant in their relationships of caregiving with the care receiver	Implement new behaviours aligned with your strengths within the context of caregiving.
	Day 15. Using your strengths	The participant	Implement new behaviours aligned with your strengths in other areas of life (personal, professional, social, etc.).
	Day 16. Sharing	The participant in their relationship with the care receiver or acquaintances regardless of the disease	Identify a person who has been a source of inspiration. Reflect on the values and lessons received.
	Day 17. Transmission	The participant in their relationship with the care receiver regardless of the disease	Reflect on the values and lessons received and how you continue to pass them on.
	Day 18. Building up strengths	The participant in their relationship with the care receiver regardless of the disease	Develop new strengths by implementing new behaviours in daily life and in your relationship with the care receiver.
Week 4	Day 19. A touch of gratitude	The participant	Experience a pleasant or satisfying moment and feel gratitude for it.
	Day 20. Gratitude	The participant	Become aware of the good things that happen to you and let a feeling of gratitude grow within you.
	Day 21. A touch of gratitude	The participant	Recall a moment filled with gratitude and recognition towards someone. Express your gratitude by writing a message of appreciation.
	Day 22. A touch of gratitude	The participant in their relationships of caregiving with the care receiver	Experience a moment of gratitude and recognition in the context of caregiving. Let this feeling of gratitude grow and internally thank the source of that moment.
	Day 23. A touch of gratitude	The participant in their relationship with the care receiver or acquaintances regardless of the disease	Experience a moment of gratitude and recognition in your relationship with the care receiver. Let this feeling of gratitude grow and internally thank the source of that moment.
	Day 24. A touch of gratitude	The participant in their relationships of caregiving with the care receiver	Recall a moment of gratitude towards someone involved in the caregiving context. Express your recognition by writing a letter of gratitude.
Week 5	Day 25. Positive words for those around you	The participant	Identify good news and experiment with techniques to receive or share good news in a way that maximizes the emotions felt.
	Day 26. Positive words for those around you	The participant in their relationship with the care receiver or acquaintances regardless of the disease	Experiment with techniques to receive or share good news with the care receiver in a way that maximizes the emotions felt.
	Day 27. Positive words for those around you	The participant	Connect techniques for receiving or sharing good news with your personal strengths.
	Day 28. Letting go of moments of frustration	The participant in their relationships of caregiving with the care receiver	Focus on a situation that caused frustration in the caregiving context and recall positive characteristics of the care receiver.
	Day 29. Forgiveness	The participant	Recall a situation where you felt hurt, identify the emotions related to forgiveness, and write a message granting forgiveness.
	Day 30. Forgiveness	The participant	Recall a situation where you think you may have hurt someone, identify the emotions related to forgiveness, and write a message asking for forgiveness.
Week 6	Day 31. Activities that have meaning	The participant	Create a list of activities and tasks completed over the past week, assess the meaning and enjoyment of these tasks.
	Day 32. Activities that have meaning	The participant	Engage in an activity that holds personal meaning from the list of activities created the previous day.
	Day 33. Using your strengths	The participant in their relationships of caregiving with the care receiver	Implement new behaviours in “challenging” situations in the caregiving context by relying on your strengths.
	Day 34. Two aspects of help: giving and receiving	The participant in their relationships of caregiving with the care receiver	Strengthen social support by reflecting on memories of giving and receiving help.
	Day 35. Changing a habit	The participant in their relationship with the care receiver or acquaintances regardless of the disease	Encourage modifying a small habit to bring more flexibility and greater positive emotions.
	Day 36. The Optimistic Self		Envision a realistic and favourable future, connect this vision to your personal strengths.
Week 7	Day 37. Codeveloping and maintaining links	The participant in their relationship with the care receiver or acquaintances regardless of the disease	Connect with the care receiver through a memory of a positive experience shared with them.
	Day 38. Shared activities	The participant in their relationship with the care receiver or acquaintances regardless of the disease	List enjoyable activities to share with the care receiver, reflecting on possible adaptations and positive alternatives.
	Day 39. Life story: past	The participant in their relationship with the care receiver or acquaintances regardless of the disease	Describe a memorable moment that strengthened the caregiver—care receiver bond.
	Day 40. Life story: present	The participant in their relationship with the care receiver or acquaintances regardless of the disease	Describe your current life with the care receiver, the challenges you face, and your shared strengths.
	Day 41. Life story: future	The participant in their relationship with the care receiver or acquaintances regardless of the disease	Formulate positive wishes for yourself and the care receiver.
	Day 42. Shared activities	The participant in their relationship with the care receiver or acquaintances regardless of the disease	Engage in a pleasant activity to share with the care receiver based on the list of activities.
Week 8	Day 43. Three little things	The participant	Notice and describe three aspects of your daily life that bring positive emotions and contribute to making your day more enjoyable.
	Day 44. Three little things	The participant in their relationship with the care receiver or acquaintances regardless of the disease	Notice and describe three aspects of your daily life that bring positive emotions, related to the care receiver.
	Day 45. Three little things	The participant in their relationships of caregiving with the care receiver	Notice and describe three aspects of your daily life that bring positive emotions related to the caregiving situation.
	Day 46. Activities that have meaning	The participant	Expand your list of enjoyable activities with new ones, and schedule two activities to do in the upcoming week.
	Day 47. Giving time to yourself	The participant	Continue dedicating time for yourself as you did during the program by engaging in meaningful and fulfilling activities.
	Day 48. Certificate of achievement	The participant	Congratulate yourself for completing the program.
